# Transformation of immunosuppressive mtKRAS tumors into immunostimulatory tumors by Nerofe and Doxorubicin

**DOI:** 10.18632/oncotarget.28467

**Published:** 2023-07-01

**Authors:** Joel Ohana, Uziel Sandler, Orly Devary, Yoram Devary

**Affiliations:** ^1^Immune System Key (ISK) Ltd., Jerusalem 9746009, Israel; ^2^Department of Bio-Informatics, Lev Academic Center (JCT), Jerusalem 91160, Israel

**Keywords:** Colorectal cancer, KRAS, apoptosis, hormone peptide, endoplasmic reticulum stress

## Abstract

Members of the rat sarcoma viral oncogene (RAS) subfamily KRAS are frequently mutated oncogenes in human cancers and have been identified in pancreatic ductal, colorectal, and lung adenocarcinomas. In this study, we show that a derivative of the hormone peptide Tumor Cell Apoptosis Factor (TCApF), Nerofe^™^ (dTCApFs), in combination with Doxorubicin (DOX) substantially reduces viability of tumor cells. It was observed that the combination of Nerofe and DOX downregulated KRAS signaling via miR217 upregulation, resulting in enhanced apoptosis of tumor cells. In addition, the combination of Nerofe and DOX also resulted in activation of the immune system against tumor cells, manifested by an increase in the immunostimulatory cytokines IL-2 and IFN-γ as well as the recruitment of NK cells and M1 macrophages to the tumor site.

## INTRODUCTION

Kirsten rat sarcoma virus (KRAS) is a member of the Rat Sarcoma Viral Oncogene (RAS) subfamily, which is a member of the RAS protein superfamily [1]. RAS proteins act as on/off switches that regulate a wide range of essential cellular processes including differentiation, proliferation, morphology, polarity, adhesion, migration, and apoptosis [2]. KRAS was discovered in 1983 by Willer and Lowy independently in human lung cancer cells [3, 4]. In proximity to this discovery, Neuroblastoma and Harvery rat sarcoma viral oncogenes (NRAS and HRAS), two KRAS paralogs, have been discovered [5–7]. KRAS, HRAS, and NRAS are oncogenes that are frequently mutated human cancers [8], with KRAS mutations being the most prevalent, forming 85% of all RAS mutations and affecting about 13% of cancer patients [9]. mtKRAS is found in many types of cancers, but the prevalence of KRAS mutations differs greatly between cancers. It has been identified in approximately 88% of pancreatic ductal adenocarcinomas (PDAC), 47% of colorectal adenocarcinomas (CRC), and 33% of lung adenocarcinomas [10, 11].

Most mutations in Ras genes are missense mutations that impair Ras GTPase, causing the Ras protein to be locked in a GTP-bound active state, thereby activating downstream oncogenic pathways. KRAS mutations occur early in tumor development, and hotspot residues G12, G13, and Q61 are highly conserved coding sequences that are the most common mutation sites [12–14].

Dysregulation of KRAS leads to tumor growth, and its signaling pathways induce modifications in the solid tumor microenvironment (TME). The downstream effector pathways of mtKRAS have been linked with decreased expression Major Histocompatibility Class I (MHC I) and increased expression of programmed cell death-ligand 1 (PD-L1). The accumulation and maintenance of myeloid-derived suppressor cells (MDSCs) in the TME has also been attributed to mtKRAS, contributing to the immunosuppressive state of the TME [15], all of which ultimately negatively affect the therapeutic response. Patients with KRAS mutated cancers generally show poor therapeutic outcome to chemotherapy, have radiation resistance, and poor prognosis [16].

Recently, two drugs that specifically target KRAS G12C, sotorasib (Lumakras^™^) and adagrasib (Krazati^™^), have received accelerated approval by the FDA for the treatment of adult patients with KRAS G12C-mutated locally advanced or metastatic NSCLC, who have received at least one prior systemic therapy [17, 18]. These drugs are the first RAS GTPase family inhibitors to be approved for clinical use, representing a major breakthrough in the field of precision oncology.

Tumor cell apoptosis factor (TCApF) is a human hormonal peptide that is naturally expressed in the thymus, colon, and frontal lobes of the brain. It was initially identified during a bioinformatics screening and found to have anti-cancer activity on cancer cell lines. As previously described, “dTCApFs (Nerofe^™^, Immune System Key Ltd., Jerusalem, Israel) is a short 14 amino acid derivative of TCApF that retains its anti-cancer activity” [19].

Studies on several cancer cell lines have revealed that “Nerofe (dTCApFs) enters cells through the T1/ST2 receptor and induces apoptosis through a novel mechanism involving the loss of Golgi function and induction of endoplasmic reticulum (ER) stress, along with downregulation of the ER stress repair mechanism” [20]. It was previously tested in a Phase I clinical trial in patients with solid tumors and was found to be “safe and potentially efficacious”. In these tumors, in addition to the induction of ER stress, treatment with Nerofe was found to suppress angiogenesis and activate the innate immune response. Specifically, Nerofe at mid-range doses of 12–48 mg/m2 reduced the expression of multiple angiogenic factors and increased the expression of anticancer cytokines in the serum of treated patients. Furthermore, a retrospective biomarker analysis showed that patients whose tumors expressed higher levels of the Nerofe receptor T1/ST2 exhibited a better response to Nerofe [21].

Nerofe’s receptor, T1/ST2, is involved in the progression of different types of cancers, including breast, colorectal, and non-small-cell lung cancer (NSCLC) [22]. It is sparsely expressed in healthy tissues but strongly overexpressed in mtKRAS tumors [23]. Of the patients who participated in the Phase I clinical trial, approximately half had colorectal cancer and one-third had pancreatic cancer. Both cancers are known to be highly mutated in KRAS [23]. Therefore, we tested whether the efficacy of Nerofe could be further enhanced by combining it with chemotherapeutic agents used in malignancies of the gastrointestinal (GI) tract (e.g., SN38, 5FU, paclitaxel, and gemcitabine). Among the combinations tested, Doxorubicin (DOX) exhibited a synergistic effect, resulting in a substantial reduction in tumor cell viability. DOX is a known inhibitor of the Unfolded Protein Response (UPR) [24], indicating a potential synergistic effect with Nerofe through ER stress induction and inhibition of the ER stress response. Moreover, low doses of DOX have also been shown to activate the adaptive immune system [25], indicating an additional level of synergy with Nerofe via the activation of both the adaptive and innate immune systems.

The objective of the present study was to investigate the synergistic effect of the combination of Nerofe and DOX in colorectal cancer and its underlying mechanism.

## RESULTS

### The combination of Nerofe and DOX has a synergistic effect on the viability and reactive oxygen species (ROS) production of metastatic colorectal cancer (mCRC) cells

**Figure 1:**
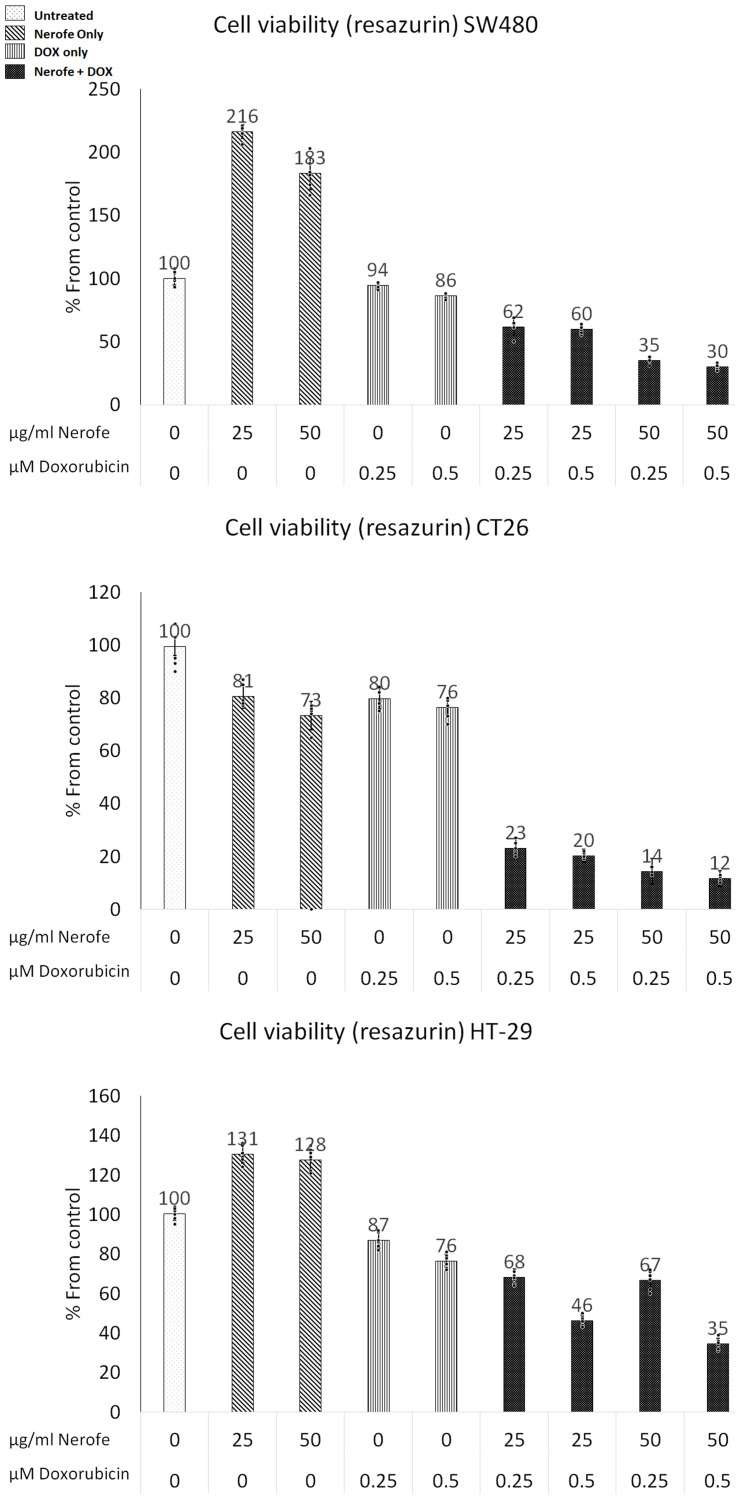
Effect of NF and DOX on the viability of mCRC cells. SW480, CT26, and HT-29 cells were treated with Nerofe (72 h) or DOX (48 h) or a combination of these. Viability was tested using a Resazurin assay kit.

The combination of Nerofe and Dox decreased the viability of metastatic colorectal cancer (mCRC) cells by 50–70% (Figure 1, Table 1 row A). Interestingly, treatment with Nerofe alone caused an apparent increase in the viability of SW480 and HT-29 cancer cells. A probable reason for this is that the viability test was performed using a resazurin agent that measures mitochondrial activity. As Nerofe causes ER stress, it increases mitochondrial activity in Nerofe-treated cells prior to apoptosis [26]. Treatment with DOX alone reduced cell viability by only 10–15%, whereas its combination with Nerofe caused a reduction in cell viability across the three different cell lines in a dose-dependent manner. The combination of Nerofe and DOX resulted in a staggering 60–80% increase in ROS production as compared to only a 60% or 10% increase following treatment with Nerofe or DOX alone, respectively (Table 1, Row B).

**Table 1:** Synergies between Nerofe and DOX Cancer cell lines (SW480, HT-29, CT26) treated with Nerofe, DOX and a combination of both

Row	Test	Nerofe	DOX	Nerofe + DOX
A	Decrease in viability	0–10%	10–15%	50–70%
B	Increase in ROS production	60%	10%	60–80%
C	Increase of miR217 expression	50%	70%	200–300%
D	Decrease of KRAS/MAPK1/2 protein	10%	25%	90–100%

A nonlinear increase in apoptosis and ROS production as well as KRAS/MAPK1/2 downregulation were detected upon DOX and Nerofe combination. Each experiment on each cell line was repeated at least 3 times.

### The combination of Nerofe and DOX downregulates MAPK signaling via miR217 upregulation

**Figure 2:**
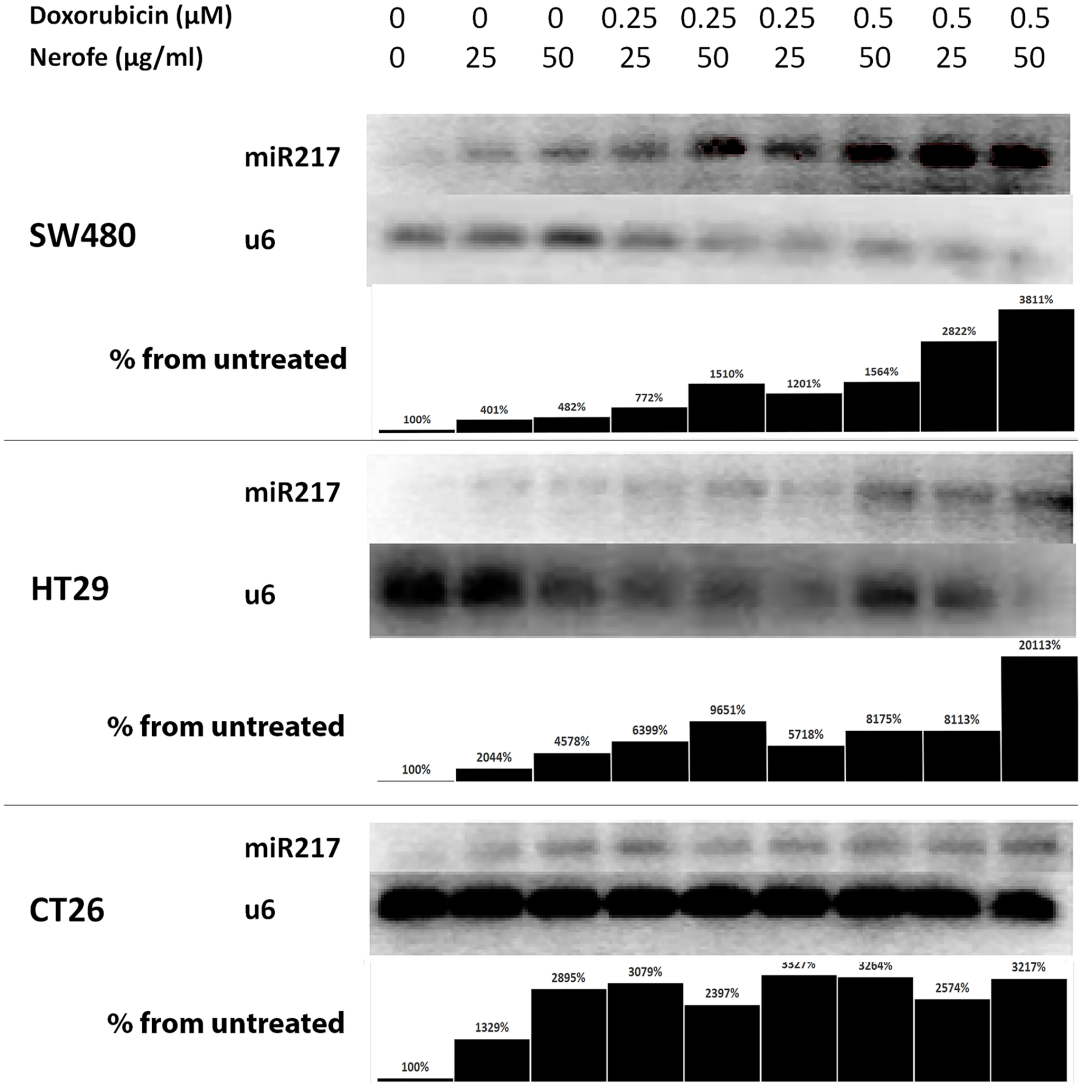
miR217 is induced by combination of Nerofe and Dox. miR217 PCR demonstrates an increase in expression following combined treatment with Nerofe and DOX. Lanes were quantified using ImageJ 1.53, and each miR217 value was normalized to its corresponding U6 value. Values are shown as the percentage of untreated sample.

**Figure 3:**
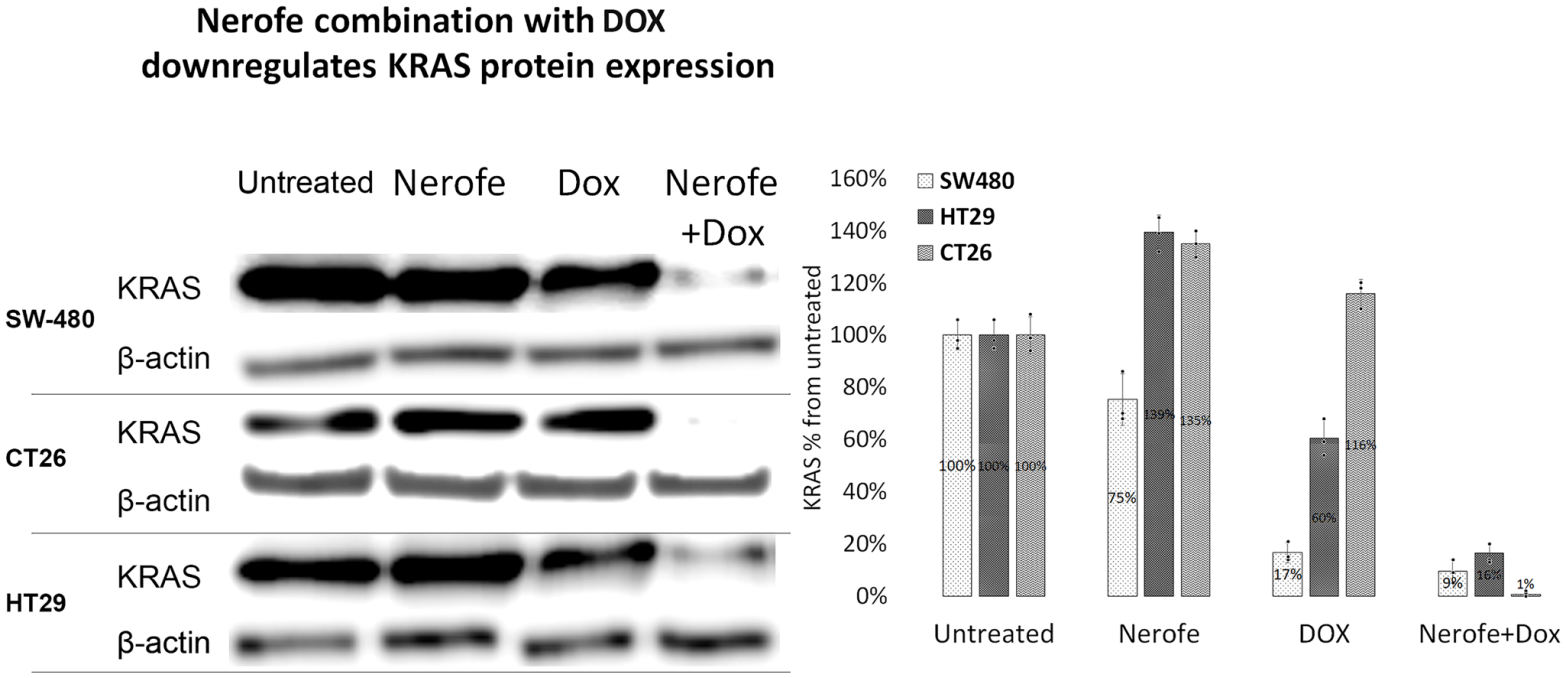
The combination of Nerofe with DOX downregulates KRAS protein expression in mCRC cell lines. SW480, CT26, and HT29 cells were treated with Nerofe, DOX, or a combination of both. Western blot analysis was performed on cell lysate. Lanes were quantified using ImageJ 1.53, and values shown are the percentage of untreated sample.

It has previously been established that the miR217 microRNA acts as a tumor suppressor in PDAC, and directly targets KRAS [27]. Additionally, bioinformatic analysis predicted that miR217 targets both KRAS and MAPK1/2 in mammalian cells (Supplementary Figure 1). To identify the potential mechanism by which the combination of Nerofe and DOX affects mCRC cell viability, the levels of miR217 were analyzed. RT-PCR analysis revealed that upon combined treatment with Nerofe and DOX, the levels of miR217 were markedly increased compared to untreated cells or cells treated with only Nerofe or DOX (Figure 2, Table 1 Row C). Indeed, KRAS protein expression levels were dramatically decreased in the three mCRC cell lines in response to Nerofe and DOX treatment (Figure 3, Table 1 Row D).

### *In vivo* studies: murine models of CRC

**Figure 4:**
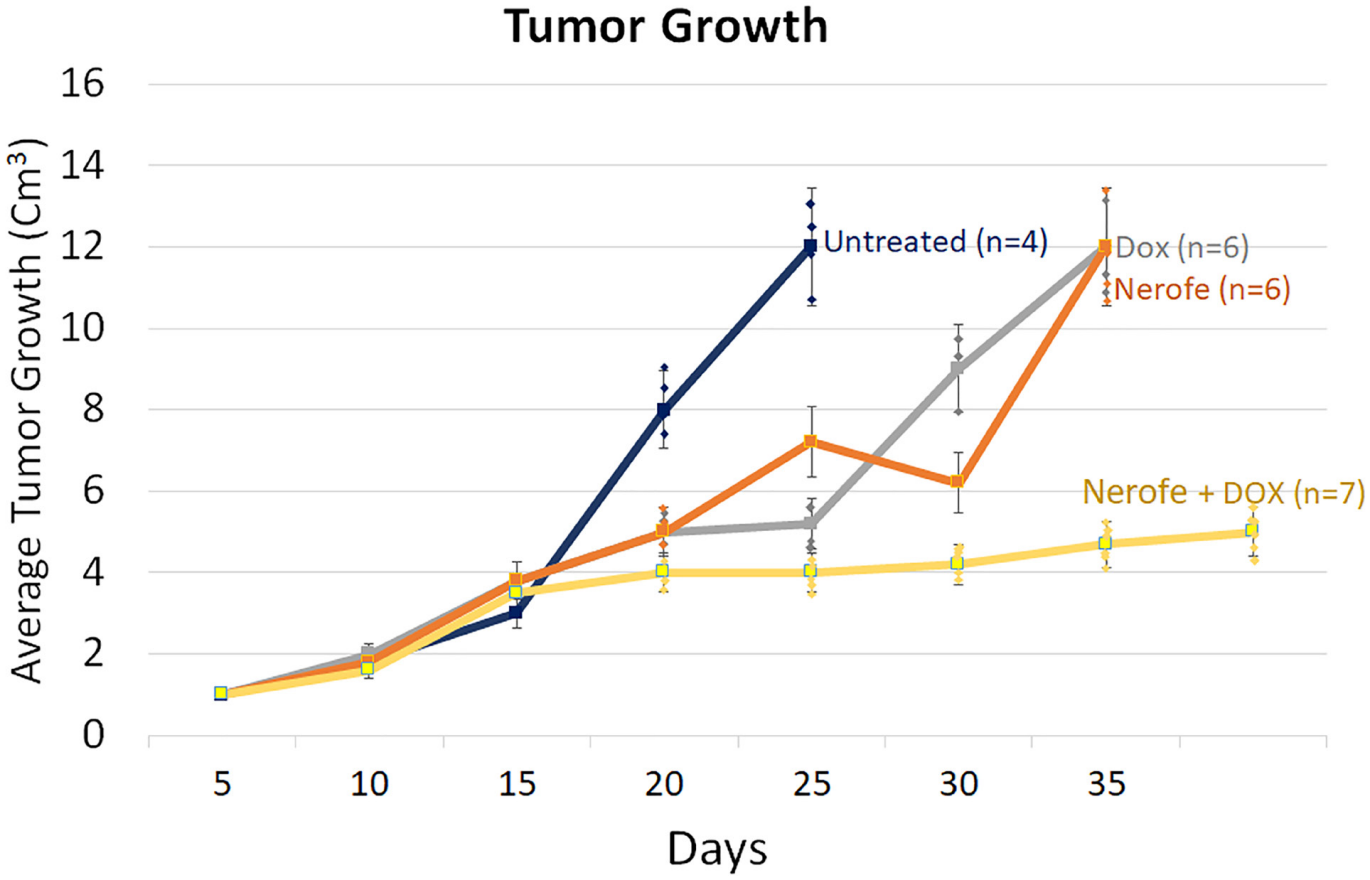
Murine model of CT26 cells (mCRC mtKRAS) inoculated subcutaneously into C57bl/6 mice. The mice were treated according to the treatments listed in the table below, and tumor size was documented.

**Figure 5:**
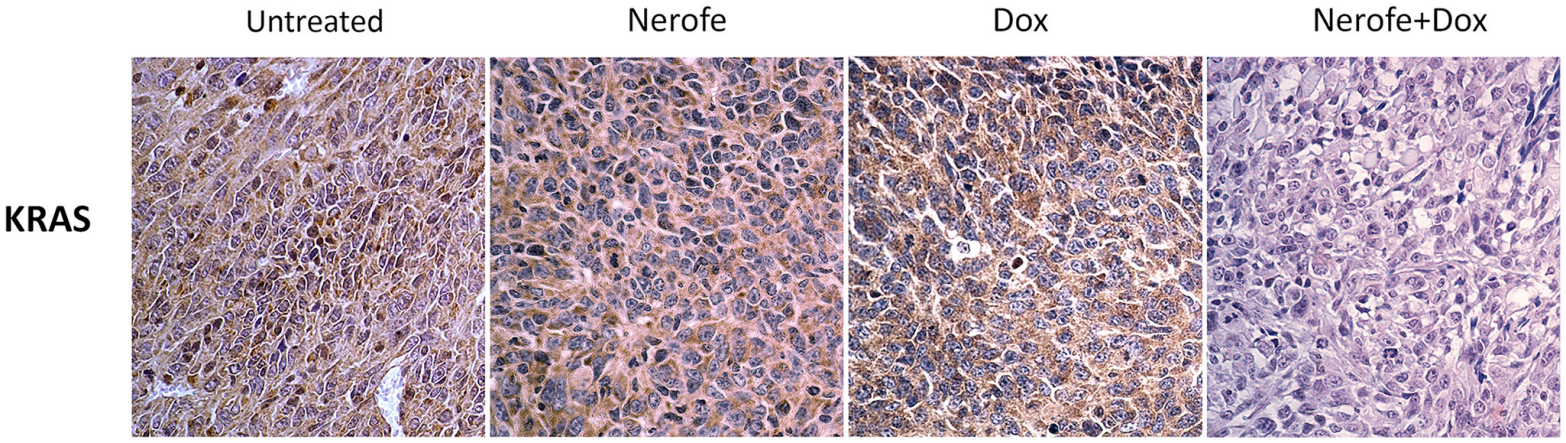
IHC analysis of murine model CT26 tumors using KRAS antibody. Brown: KRAS, Blue: Nuclear staining.

**Figure 6:**
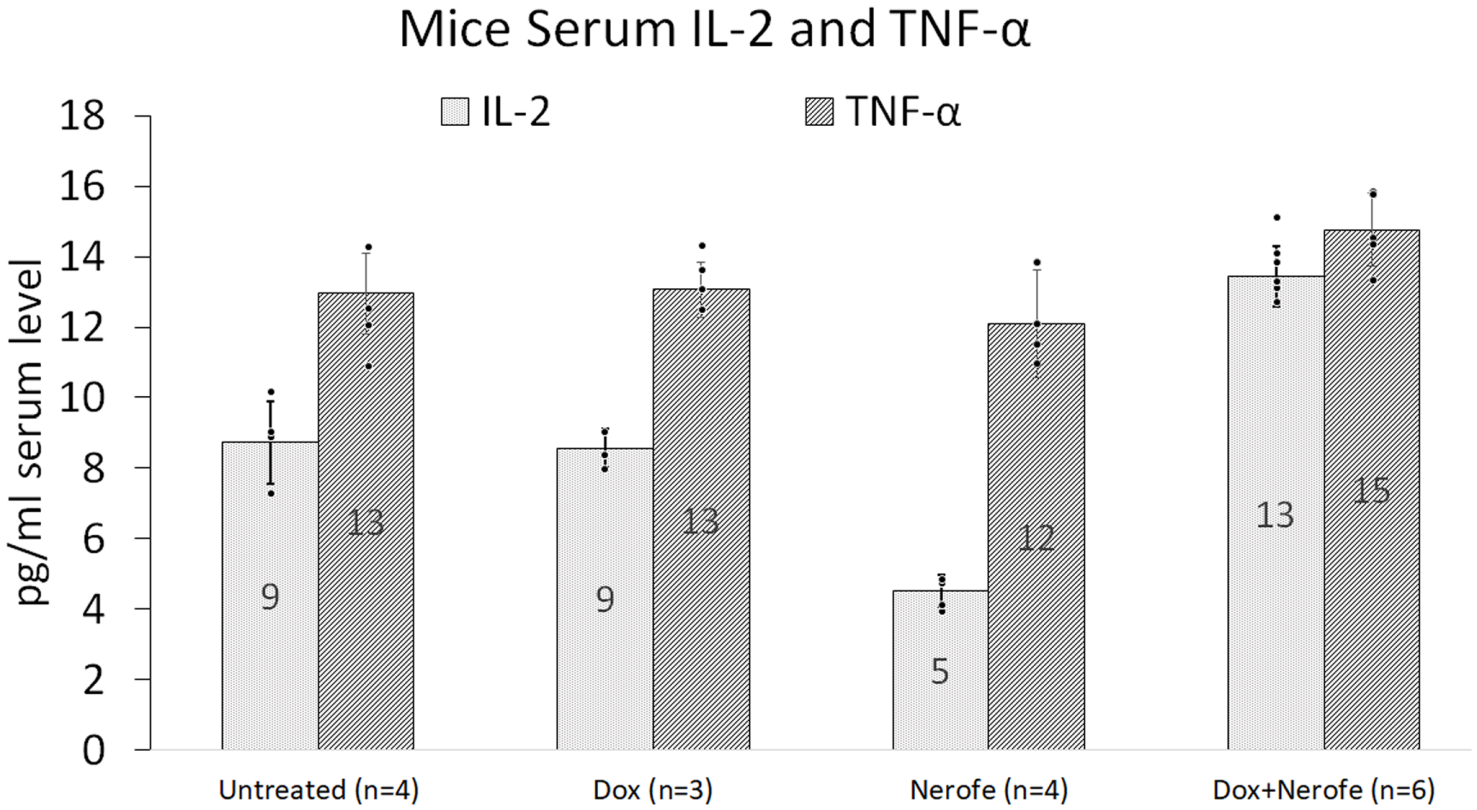
Blood cytokine profile of the mouse model. CT26 cells (mCRC mtKRAS) were inoculated subcutaneously into C57bl/6 mice, and the mice were treated with Nerofe, DOX, or a combination of both. Blood was drawn and the levels of anti-cancer immuno-cytokines IL-2 and TNF-α were analyzed in a multiplex assay using the MILLIPLEX MAP Mouse High Sensitivity T Cell Panel (*n* = 3–6).

*In vivo* experiments were performed to verify the cell culture findings. A model of CT26 cells injected into C57BL/6 mice was utilized. The mice were treated with Nerofe, DOX, or a combination of both. In response to the combined treatment, the average tumor size considerably reduced compared to the untreated group or administration of Nerofe or DOX alone (Figure 4). Like the cell culture findings, immunohistochemistry (IHC) analysis of CT26 tumors demonstrated a vast decrease in KRAS levels in response to combined treatment with Nerofe and DOX (Figure 5). Moreover, cytokine profile analysis revealed elevated IL-2 levels in the blood of mice treated with a combination of Nerofe and DOX, while TNF-*α* levels were not altered in response to any of the treatments (Figure 6).

**Table:** 

	Untreated (Blue)	DOX (Gray)	Nerofe (Orange)	Nerofe+DOX (Yellow)
*P*-value	*P* < 0.005	*P* < 0.0001	*P* < 0.007	*P* < 0.002
Correlation Coefficient	*R* = 0.99	*R* = 0.98	*R* = 0.95	*R* = 0.93
Quadric regression curve V – Tumor fold volume X – time (days)	V = 0.0044 X2 − 0.0976 X + 1.0912	V = 0.0036 X2 + 0.1984 X + 0.7705	V = 0.0073 X2 + 0.2580 X + 0.5462	V = −0.0071 X2 + 0.3270 X − 0.5228

**Figure 7:**
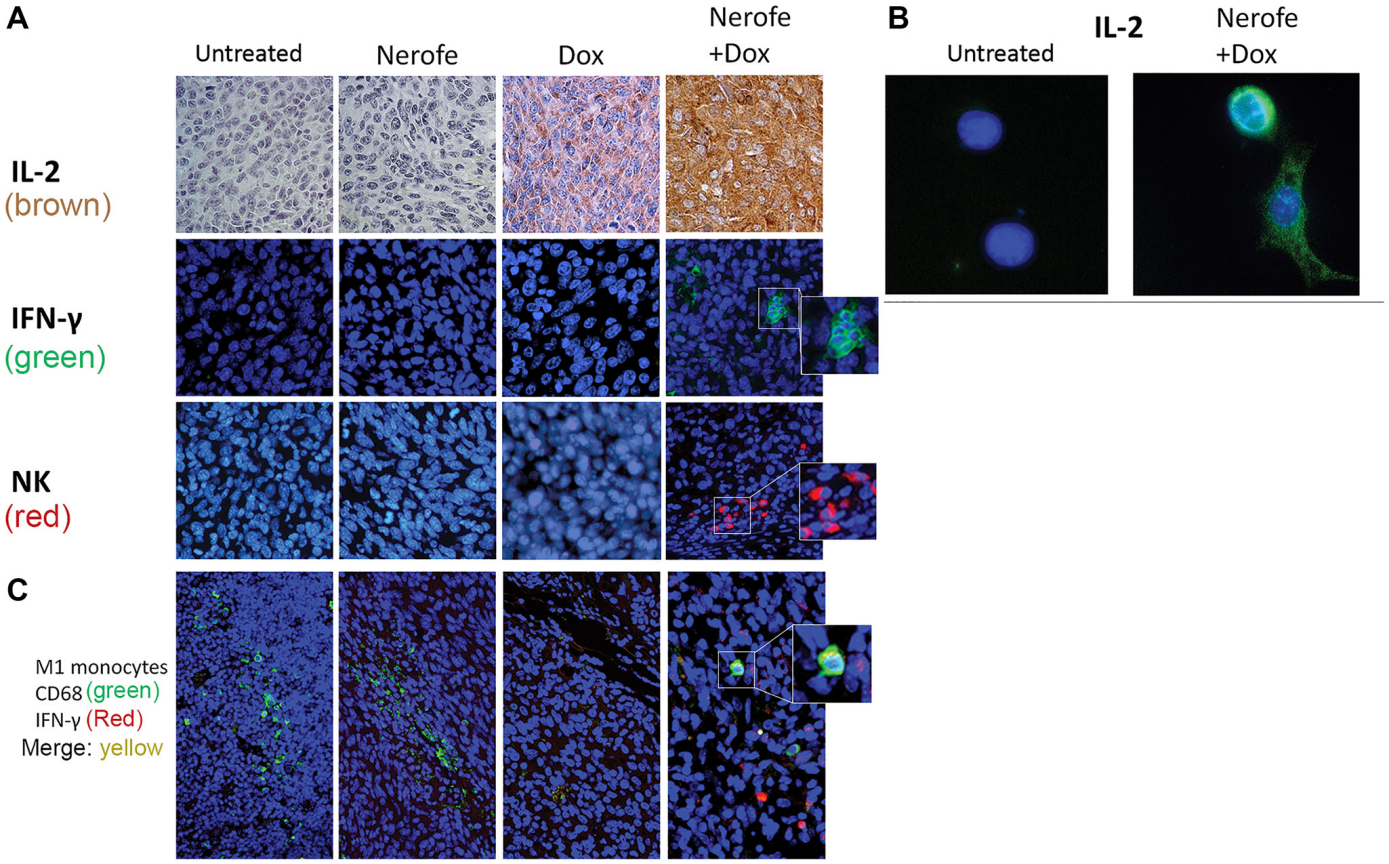
Immunological effect of Nerofe and DOX: CT26 murine model showing the immunological effect of Nerofe, DOX, and their combined treatment. (**A**) IHC of CT26 mice tumor sections stained with anti-IL-2 (brown), IFN-γ (green), and NK cell markers (red). (**B**) IF of CT26 cells stained with anti-IL-2 (green). (**C**) Treated CT26 murine model demonstrating recruitment of M1 monocytes to tumors treated with a combination of Nerofe and DOX: IHC staining of CT26 mice tumor sections with M1 monocyte marker, anti CD68 (green), and IFN-γ (red). In all panels nucleus is stained in blue.

IHC analysis of CT26 tumors further validated the elevated IL-2 levels (Figure 7A). The induction of IL-2 expression following combined treatment with Nerofe and DOX was validated *in vitro* using IF (Figure 7B). In addition, IHC analysis of the CT26 tumors demonstrated an increase in INF-*γ* levels, NK cells (Figure 7A), and M1 monocytes (Figure 7C) following combined treatment with Nerofe and DOX.

## DISCUSSION

In this study, we demonstrated that combined treatment with Nerofe and DOX has a synergistic effect that results in the downregulation of KRAS, enhanced apoptosis of cancer cells, and activation of the immune system inside the tumor. We showed that the direct cellular effect of the combination of Nerofe and DOX on mCRC resulted in reduced cell viability (Table 1, Row A and Figure 1) and increased ROS production (Table 1, Row B). We previously showed that Nerofe is concentrated in the Golgi apparatus and causes its destruction, thereby inducing ER stress. In parallel, Nerofe downregulates sXBP1, which is a central transcription factor in the activation of ER stress response [28], thus inhibiting the ER stress repair mechanism and resulting in apoptosis [2]. Similarly, DOX has been demonstrated to inhibit the IRE1α-XBP1 axis of UPR5 [5]. Therefore, the combination of Nerofe and DOX likely collectively induces ER stress and inhibits the ER stress response mechanism and the UPR, resulting in increased apoptosis of cancer cells.

Colorectal tumor growth and apoptosis were previously shown to be regulated by miR217, which targets the MAPK signaling pathway [10]. We found that combined treatment with Nerofe and DOX resulted in the induction of miR217 (Table 1, Row C and Figure 2). These *in vitro* results were confirmed *in vivo* using a murine model, which demonstrated that combined treatment with Nerofe and DOX resulted in reduced tumor size (Figure 4) along with the downregulation of KRAS expression in the tumor cells (Figure 5).

The *in vivo* model revealed that in addition to a direct effect on tumor cell apoptosis, the combined treatment of Nerofe and DOX also resulted in the activation of the immune system against the tumor. Specifically, we observed an increase in the levels of immunostimulatory cytokines IL-2 (Figures 6, 7A, and 7B) and IFN-γ (Figure 7A), as well as the recruitment of NK cells and M1 macrophages to the tumor site (Figure 7C).

It has been previously reported in a Phase I clinical trial that Nerofe increases the levels of NK cell activators (IL-21 and IL12p70) and dendritic cell (DC) activators (IL-2 and GM-CSF). This effect was accompanied by the recruitment of NK and DC cells to the tumors, indicating that Nerofe treatment activates the innate immune response [21]. DOX has previously been associated with myeloid-derived suppressor cell (MDSC) depletion and the induction of immunogenic death [11]. Moreover, the recently published results of a clinical trial conducted in patients with triple-negative breast cancer (TNBC) demonstrated that low levels of DOX resulted in tumor microenvironment alterations that enhanced the sensitivity of the tumor to anti-PD-1 and PD-L1 treatments. Low levels of DOX increase T-cell infiltration and T-cell receptor (TCR) diversity in tumors, which demonstrate the effect of DOX on the adaptive immune response [6]. Taken together, these findings indicate that a combination of Nerofe and DOX could result in the combined activation of the innate and adaptive immune systems (Supplementary Figure 2).

It was recently shown that tumor associated DCs (tDCs) exhibit ER stress and increased IRE/XBP1 activation, resulting in reduced antitumor activity. Silencing of XBP1 in tDC results in enhanced T cell anti-tumor immunity [29]. Therefore, combined treatment with Nerofe and DOX may contribute directly to DC activation in the tumor microenvironment, in addition to their effect on IL-2 production.

An additional level of synergy between Nerofe and DOX may be related to C/EBP homologous protein (CHOP), which is upregulated during ER stress [30]. Cancer cells that do not express CHOP are resistant to DOX [31]; however, Nerofe induces ER stress and CHOP expression.

The leading molecules that downregulate KRAS are Adagrasib [17], which targets G12C mutated KRAS in NSCLC, and Sotorasib (AMG-510) [18], which targets the same mutation in lung cancer.

In clinical trials, sotorasib and adagrasib have produced response rates of up to 40% and 30% in NSCLC patients, respectively. They have also been shown to improve overall survival in patients with KRAS-mutated NSCLC.

However, these drugs are not without limitations and challenges. First, not all patients with KRAS G12C-mutated NSCLC respond to these drugs, and some may develop resistance over time [32]. Second, these drugs are not effective against other KRAS variants, such as KRAS G12D, which are also prevalent in various cancers [33]. Third, they may have adverse effects on normal tissues that express wild-type KRAS, such as hepatotoxicity, renal impairment, and QTc interval prolongation [34]. It was also shown that tumor cells that were treated with specific G12C inhibitors had an initial suppression of the MAPK pathway, but afterwards adapted by activating wild RAS, thus despite continuous inhibition of GTP-bound KRAS, the MAPK pathway is reactivated within 24–48 hours [35]. Furthermore, novel mutations can bypass inhibition while retaining the initial subgroup’s inactivated state [36] and the presence of multiple concurrent mutations can result in resistance to therapy [37]. Therefore, there is still an unmet need for developing more potent and selective KRAS inhibitors that can overcome these challenges and improve the outcomes of patients with KRAS-mutated cancers.

In contrast to these limitations, combined treatment with Nerofe and DOX downregulates KRAS and inhibits the MAPK pathway regardless of the KRAS mutation. Furthermore, we have shown that following Nerofe and DOX treatment, the TME is transformed from immunosuppressive to immunostimulatory, an effect that has not been demonstrated in any of the KRAS inhibitors to date.

In conclusion, we demonstrated that the combination of Nerofe and DOX exerts a synergistic effect during mCRC treatment, which could stem from several independent and complementary mechanisms of action.

## MATERIALS AND METHODS

### Chemicals and special reagents

Nerofe^™^ (dTCApFs). dTCApFs was synthesized like previously described [19].

The following chemotherapeutic agents were also used: Doxorubicin hydrochloride (D1515) (Dox; Sigma-Aldrich, US), SN38 (H0165) (Sigma-Aldrich), 5FU (F6627) (Sigma-Aldrich), paclitaxel (T7402) (Sigma-Aldrich) and gemcitabine (G6423) (Sigma-Aldrich).

### Cell lines

Human cell lines including HT29 (ATCC^®^ HTB-38^™^), SW480 (ATCC^®^ CCL-228^™^), Panc-1 (ATCC^®^ CRL-1469^™^), and MIA PaCa-2 (ATCC^®^ CRM-CRL-1420^™^) were obtained from ATCC (USA) and used within six months. CT26 cells were kindly provided by Prof. Lea Eisenbach of the Weizmann Institute of Science. All cell lines, except CT26, were maintained in growth media containing DMEM (Gibco, Thermo Fisher Scientific, USA) supplemented with 10% fetal bovine serum (FBS; Biological Industries, Israel), 1 mM sodium pyruvate (Biological Industries), 100 units/ml penicillin and 100 μg/ml streptomycin (Biological Industries), and 250 ng/ml Amphotericin B (Biological Industries). CT26 was maintained in growth media containing RPMI 1640 (Gibco, Thermo Fisher Scientific) with 10% FBS, 1 mM sodium pyruvate, 100 units/ml penicillin and 100 μg/ml streptomycin, and 250 ng/ml Amphotericin B. All cells were maintained at 37°C in a humidified atmosphere containing 5% CO_2_.

### Cell viability assays

Cell viability assays were performed using the Resazurin Assay Kit (Abcam, Cambridge, US) on Panc-1, MIA PaCa-2, SW480, and HT29 cell lines. Cells were seeded (4000 cells/well) in 96-well black microplates with clear bottom (Greiner, US) in 100 μl cell growth media (see above) and incubated overnight. Media were then changed to 100 μl growth media supplemented with 5% D-Mannitol (Sigma-Aldrich). Cells were treated with Nerofe (25 μg/ml or 50 μg/ml) for 72 h and/or DOX (0.25 μM) for 48 h or left untreated. The percentage of viable cells was calculated and compared with that of the untreated cells.

### Polymerase chain reaction (PCR) analysis

Cells were cultured in six-well plates (NUNC) (Thermo Fisher Scientific) in growth media. After 24 h, treatment medium supplemented with 5% D-Mannitol (Sigma-Aldrich) was added as described above for the indicated time period. After the final incubation, the cells were washed with phosphate-buffered saline (PBS) and RNA was extracted using an RNeasy Mini Kit (Qiagen, Germany). The cells were then lysed directly in the wells. cDNA was prepared from RNA using a qPCRBIO cDNA Synthesis Kit (PCR Biosystems, UK). PCR was performed using GoTaq Green Master Mix (Promega, USA) (40 cycles) with the following primers: U6: F: 5′-CTC GCT TCG GCA GCA CA-3′, R: 5′-AAC GCT TCA CGA ATT TGC GT-3′; miR217: F: 5′TAC TCA ACT CAC TAC TGC ATC AGG A-3′, R: 5′-TAT GGT TGT TCT GCT CTC TGT GTC-3′. The PCR products were loaded onto 2% agarose gel and visualized using a UV CCD camera (Bio-Rad, USA). Each experiment was repeated at least three times.

### Dihydroethidium (DHE) assay

ROS levels were determined using the DHE assay as previously described (2). Cells were seeded (20,000/well) into black 96-well plates (Greiner) with 100 μl growth media overnight. Cells were then treated with Nerofe (25 μg/ml or 50 μg/ml) for 72 h and/or DOX (0.25 uM or 0.5 uM) for 48 h in treatment medium containing 5% D-Mannitol (Sigma-Aldrich). Following treatment, cells were incubated for 20 min with growth media containing 10 μM DHE and then washed three times with PBS. The plates were analyzed using a fluorescence spectrophotometer (BioTek Synergy HT; BioTek Instruments, USA) at an excitation/emission wavelength pair of 530/590 nm. All experiments were performed in triplicates.

### Bioinformatic analysis

Prediction of miR217 targets in KRAS was performed using TargetScan [38].

### Western blotting

For western blot analysis, cells were seeded (1.8 M cells/75 cm^2^ flask) in 30 ml growth medium and incubated overnight. Cells were then treated with Nerofe (25 μg/ml or 50 μg/ml) twice for 4 and 6 d and/or DOX (0.25 μM or 0.5 μM) for 3 d in treatment medium, which was composed of the growth medium supplemented with 5% Mannitol (Sigma-Aldrich). At the end of the treatment, the cells were washed twice with PBS and scraped from the flask and into PBS using a cell scraper. Cells were centrifuged for 10 min at 300 × g and 4°C, and the pellet was lysed with lysis buffer containing RIPA Buffer (Sigma-Aldrich), protease inhibitor (Halt^™^ Protease Inhibitor Cocktail, Thermo Fisher Scientific), phosphatase inhibitors (Sigma-Aldrich), and 25 U/ml Benzonase^®^ Nuclease (Purity > 90%, Millipore, USA) and incubated for 20 min on ice. A reducing agent (Invitrogen, USA) was added to each sample and incubated for 10 min on ice. The supernatant was collected after centrifuging the cells at 15,000 × g for 15 min at 4°C, and LDS sample buffer (Invitrogen) was added to each sample. The samples were heat-denatured for 5 min and loaded onto–4–20% Tris-Glycine precast gels (NuSep, USA). Western blotting was performed using a semi-dry wet transfer device with Tris-Glycine transfer buffer (Bio-Rad) onto a PVDF membrane (Millipore). The membrane was blocked for one hour using 5% skim milk in TBST (Amresco, USA). Anti-KRAS (WH0003845M1) (Sigma-Aldrich) was used as the primary antibody, diluted 1:500 in 5% skim milk in TBST, and incubated with shaking overnight at 4°C. The secondary antibody was Anti-mouse IgG, HRP-linked #7076 (Cell Signaling Technology, USA), diluted 1:2000 in 5% skim milk in TBST. ECL was developed using SuperSignal West Femto Maximum Sensitivity Substrate (Thermo Fisher Scientific) using an LI-COR C-Digit scanner, and quantification was performed using Image studio 5.2.

### Murine model of mtKRAS mCRC

C57BL/6 mice (age: 5 weeks; weight: 20 g) were acquired from Harlan Laboratories Ltd. (Israel). Mice were subcutaneously injected with 100,000 CT26 cells (metastatic colorectal cancer, KRAS-mutated; mCRC mtKRAS). Nerofe and/or DOX treatment commenced after the tumor size reached 50 mm^3^ at its largest dimension (7–10 d after injection). Mice were divided into four different groups of 3–6 mice: Group 1–untreated group, injected with 5% Mannitol IP once a week; Group 2–treated with Nerofe (15 mg/kg, once a week); Group 3–treated with DOX (2 mg/kg, once a week); and Group 4–treated with a combination of Nerofe (15 mg/kg, once a week) +DOX (2 mg/kg on the same day as Nerofe).

During treatment, the tumor size was documented. At the end of treatment, the mice were euthanized, serum and tumors were collected like previously described [20].

### Cytokine profile of mice blood samples

Blood was drawn from each mouse and collected separately in a 1.5 ml vial. After 30 min to 1 h of incubation at room temperature ranging between 20°C and 25°C (RT), the clotted blood was centrifuged at 300 × g for 15 min at 4°C. The supernatant was collected, centrifuged, and frozen at −70°C for later analysis. Prior to analysis, the blood was quickly thawed in a water bath at RT and then centrifuged at 13,000 × g for 10 min at 4°C. The levels of anti-cancer immunocytokines IL-2 and TNF-α were analyzed using a multiplex assay according to MHSTCMAG-70K guidelines of the Milliplex Map Mouse High Sensitivity T Cell Panel (Millipore). Plates were read using a Bio-Plex 200 reader (Bio-Rad).

### IHC (chromogenic/fluorescent)

IHC analyses of the paraffin-embedded tumor sections were performed like previously described [20] with minor changes: “deparaffinizing the sections with xylene washes and rehydrating them with serial washes of absolute ethanol, 95% ethanol, and distilled water. Antigen retrieval was performed by heating the sections in 10 mM sodium citrate buffer (pH 6.0) to temperatures up to 100°C. After staining with HRP secondary antibodies, endogenous peroxidase was blocked with 3% oxygen peroxidase for 10 min. Non-specific peptide binding was blocked using Background Buster (Innovex Biosciences, USA) for 30 min at RT.” Primary antibodies were diluted with 100-400 μl SignalStain Antibody Diluent (Cell Signaling Technology) per slide and incubated overnight at 4°C. For chromogenic HRP staining, secondary antibody binding and 3,3’-diaminobenzidine (DAB) development were performed the following day using ImmPACT^®^ DAB Substrate, Peroxidase (HRP) (SK-4105) (Vector Laboratories, USA). Nuclei were stained with hematoxylin according to the manufacturer’s instructions (Sigma-Aldrich). Slides stained with fluorescent secondary antibodies were subsequently stained with DAPI (1 μg/ml, Sigma-Aldrich). The slides were dehydrated, mounted using FluoreGuard Mounting Medium (Hard Set) (ScyTek Laboratories, USA), and visualized under an Olympus BX41 microscope (Olympus, Japan) with a CCD camera. Primary antibodies used were: Anti-KRAS antibody (Abcam ab216890, diluted 1:100), p44/42 MAPK (Erk1/2) (137F5) Rabbit mAb (Cell signaling 4695, diluted 1:250), Anti-IL-2 antibody (Abcam ab180780, diluted 1:50), Anti-Interferon gamma antibody (Abcam ab9657, diluted 1:400), Anti-CD68 antibody (KP1) (ab955, diluted 1:100), and NK1.1 Monoclonal Antibody (Biolegend PK136, diluted 1:100). For chromogenic HRP, the secondary antibodies used were ImmPRESS^®^ HRP Goat Anti-Rabbit IgG Polymer Detection Kit Peroxidase and ImmPRESS^®^ HRP Goat Anti-Mouse IgG Polymer Detection Kit (Peroxidase) (Vector Laboratories). Secondary antibodies for fluorescent staining were: Anti-rabbit IgG (H+L), F(ab’)2 Fragment (Alexa Fluor^®^ 488 Conjugate) (Cell Signaling), Anti-mouse IgG (H+L), F(ab’)2 Fragment (Alexa Fluor^®^ 555 Conjugate) (Cell Signaling).

### ImmunoCytoChemistry (ICC)

CT26 cells were seeded (15,000 cells/well) on a chamber slide (Nunc^™^ Lab-Tek^™^ II Chamber Slide System, Thermo Fisher Scientific) in 1 ml growing medium and cultured overnight. On the following day, the cells were treated with 50 μg/ml Nerofe and/or 0.5 μM DOX for 72 h in the treatment medium, which was composed of the growth medium supplemented with 5% Mannitol (Sigma-Aldrich). Cells were fixed with 4% formaldehyde in PBS for 15 min at RT, permeabilized with 0.25% Triton X-100 (Amresco) in PBS for 10 min at RT, and blocked for 1 h with 1% bovine serum albumin (BSA) (Sigma-Aldrich) in PBS along with 22.52 mg/mL glycine (Sigma-Aldrich) and 0.1% Tween-20 (Amresco). Cells were incubated overnight at 4°C with the primary antibody Anti-IL2 antibody (ab180780, Abcam) diluted 1:100 with SignalStain Antibody Diluent (Cell Signaling Technology). The following day, cells were stained for 1 h with the secondary antibody anti-rabbit IgG (H+L), F(ab’)2 Fragment Alexa Fluor^®^ 488 Conjugate (Cell Signaling Technology), diluted 1:1000 with Immunofluorescence Antibody Dilution Buffer (Cell Signaling Technology), and the nuclei were stained with DAPI. Finally, the wells were removed from the slides, mounted with Aqueous Mounting Medium (ab128982, Abcam), and visualized under a fluorescent microscope at X40 and X100 magnifications (Olympus BX41).

## SUPPLEMENTARY MATERIALS



## References

[1] Colicelli J . Human RAS superfamily proteins and related GTPases. Sci STKE. 2004; 2004:RE13. 10.1126/stke.2502004re13. 15367757 PMC2828947

[2] Wennerberg K , Rossman KL , Der CJ . The Ras superfamily at a glance. J Cell Sci. 2005; 118:843–46. 10.1242/jcs.01660. 15731001

[3] Shimizu K , Goldfarb M , Suard Y , Perucho M , Li Y , Kamata T , Feramisco J , Stavnezer E , Fogh J , Wigler MH . Three human transforming genes are related to the viral ras oncogenes. Proc Natl Acad Sci U S A. 1983; 80:2112–16. 10.1073/pnas.80.8.2112. 6572964 PMC393767

[4] McCoy MS , Toole JJ , Cunningham JM , Chang EH , Lowy DR , Weinberg RA . Characterization of a human colon/lung carcinoma oncogene. Nature. 1983; 302:79–81. 10.1038/302079a0. 6298638

[5] Barbacid M . ras genes. Annu Rev Biochem. 1987; 56:779–827. 10.1146/annurev.bi.56.070187.004023. 3304147

[6] Chang EH , Gonda MA , Ellis RW , Scolnick EM , Lowy DR . Human genome contains four genes homologous to transforming genes of Harvey and Kirsten murine sarcoma viruses. Proc Natl Acad Sci U S A. 1982; 79:4848–52. 10.1073/pnas.79.16.4848. 6289320 PMC346782

[7] McBride OW , Swan DC , Tronick SR , Gol R , Klimanis D , Moore DE , Aaronson SA . Regional chromosomal localization of N-ras, K-ras-1, K-ras-2 and myb oncogenes in human cells. Nucleic Acids Res. 1983; 11:8221–36. 10.1093/nar/11.23.8221. 6672765 PMC326577

[8] Soh J , Okumura N , Lockwood WW , Yamamoto H , Shigematsu H , Zhang W , Chari R , Shames DS , Tang X , MacAulay C , Varella-Garcia M , Vooder T , Wistuba II , et al. Oncogene mutations, copy number gains and mutant allele specific imbalance (MASI) frequently occur together in tumor cells. PLoS One. 2009; 4:e7464. 10.1371/journal.pone.0007464. 19826477 PMC2757721

[9] Simanshu DK , Nissley DV , McCormick F . RAS Proteins and Their Regulators in Human Disease. Cell. 2017; 170:17–33. 10.1016/j.cell.2017.06.009. 28666118 PMC5555610

[10] Huang L , Guo Z , Wang F , Fu L . KRAS mutation: from undruggable to druggable in cancer. Signal Transduct Target Ther. 2021; 6:386. 10.1038/s41392-021-00780-4. 34776511 PMC8591115

[11] Prior IA , Hood FE , Hartley JL . The Frequency of Ras Mutations in Cancer. Cancer Res. 2020; 80:2969–74. 10.1158/0008-5472.CAN-19-3682. 32209560 PMC7367715

[12] Bourne HR , Sanders DA , McCormick F . The GTPase superfamily: a conserved switch for diverse cell functions. Nature. 1990; 348:125–32. 10.1038/348125a0. 2122258

[13] Bourne HR , Sanders DA , McCormick F . The GTPase superfamily: conserved structure and molecular mechanism. Nature. 1991; 349:117–27. 10.1038/349117a0. 1898771

[14] Scheffzek K , Ahmadian MR , Kabsch W , Wiesmüller L , Lautwein A , Schmitz F , Wittinghofer A . The Ras-RasGAP complex: structural basis for GTPase activation and its loss in oncogenic Ras mutants. Science. 1997; 277:333–38. 10.1126/science.277.5324.333. 9219684

[15] van Maldegem F , Downward J . Mutant KRAS at the Heart of Tumor Immune Evasion. Immunity. 2020; 52:14–16. 10.1016/j.immuni.2019.12.013. 31951548

[16] Mak RH , Hermann G , Lewis JH , Aerts HJ , Baldini EH , Chen AB , Colson YL , Hacker FH , Kozono D , Wee JO , Chen YH , Catalano PJ , Wong KK , Sher DJ . Outcomes by tumor histology and KRAS mutation status after lung stereotactic body radiation therapy for early-stage non-small-cell lung cancer. Clin Lung Cancer. 2015; 16:24–32. 10.1016/j.cllc.2014.09.005. 25450872 PMC4427190

[17] FDA grants accelerated approval to adagrasib for KRAS G12C-mutated NSCLC. U.S. Food & Drug. 2022. https://www.fda.gov/drugs/resources-information-approved-drugs/fda-grants-accelerated-approval-adagrasib-kras-g12c-mutated-nsclc.

[18] FDA grants accelerated approval to sotorasib for KRAS G12C-mutated NSCLC. U.S. Food & Drug. 2021. https://www.fda.gov/drugs/resources-information-approved-drugs/fda-grants-accelerated-approval-sotorasib-kras-g12c-mutated-nsclc.

[19] Sandler U , Devary O , Braitbard O , Ohana J , Kass G , Rubinstein AM , Friedman ZY , Devary Y . NEROFE--a novel human hormone-peptide with anti-cancer activity. J Exp Ther Oncol. 2010; 8:327–39. 21222365

[20] Ohana J , Sandler U , Kass G , Stemmer SM , Devary Y . dTCApFs, a derivative of a novel human hormone peptide, induces apoptosis in cancer cells through a mechanism involving loss of Golgi function. Mol Clin Oncol. 2017; 7:991–99. 10.3892/mco.2017.1453. 29285362 PMC5740848

[21] Stemmer SM , Benjaminov O , Silverman MH , Sandler U , Purim O , Sender N , Meir C , Oren-Apoteker P , Ohana J , Devary Y . A phase I clinical trial of dTCApFs, a derivative of a novel human hormone peptide, for the treatment of advanced/metastatic solid tumors. Mol Clin Oncol. 2018; 8:22–29. 10.3892/mco.2017.1505. 29423221 PMC5772927

[22] Hong J , Kim S , Lin PC . Interleukin-33 and ST2 Signaling in Tumor Microenvironment. J Interferon Cytokine Res. 2019; 39:61–71. 10.1089/jir.2018.0044. 30256696 PMC6350413

[23] Tago K , Ohta S , Kashiwada M , Funakoshi-Tago M , Matsugi J , Tominaga SI , Yanagisawa K . ST2 gene products critically contribute to cellular transformation caused by an oncogenic Ras mutant. Heliyon. 2017; 3:e00436. 10.1016/j.heliyon.2017.e00436. 29226265 PMC5714553

[24] Jiang D , Lynch C , Medeiros BC , Liedtke M , Bam R , Tam AB , Yang Z , Alagappan M , Abidi P , Le QT , Giaccia AJ , Denko NC , Niwa M , Koong AC . Identification of Doxorubicin as an Inhibitor of the IRE1α-XBP1 Axis of the Unfolded Protein Response. Sci Rep. 2016; 6:33353. 10.1038/srep33353. 27634301 PMC5025885

[25] Voorwerk L , Slagter M , Horlings HM , Sikorska K , van de Vijver KK , de Maaker M , Nederlof I , Kluin RJC , Warren S , Ong S , Wiersma TG , Russell NS , Lalezari F , et al. Immune induction strategies in metastatic triple-negative breast cancer to enhance the sensitivity to PD-1 blockade: the TONIC trial. Nat Med. 2019; 25:920–28. 10.1038/s41591-019-0432-4. 31086347

[26] Bravo R , Gutierrez T , Paredes F , Gatica D , Rodriguez AE , Pedrozo Z , Chiong M , Parra V , Quest AF , Rothermel BA , Lavandero S . Endoplasmic reticulum: ER stress regulates mitochondrial bioenergetics. Int J Biochem Cell Biol. 2012; 44:16–20. 10.1016/j.biocel.2011.10.012. 22064245 PMC4118286

[27] Zhao WG , Yu SN , Lu ZH , Ma YH , Gu YM , Chen J . The miR-217 microRNA functions as a potential tumor suppressor in pancreatic ductal adenocarcinoma by targeting KRAS. Carcinogenesis. 2010; 31:1726–33. 10.1093/carcin/bgq160. 20675343

[28] Jiang D , Niwa M , Koong AC . Targeting the IRE1α-XBP1 branch of the unfolded protein response in human diseases. Semin Cancer Biol. 2015; 33:48–56. 10.1016/j.semcancer.2015.04.010. 25986851 PMC4523453

[29] Cubillos-Ruiz JR , Silberman PC , Rutkowski MR , Chopra S , Perales-Puchalt A , Song M , Zhang S , Bettigole SE , Gupta D , Holcomb K , Ellenson LH , Caputo T , Lee AH , et al. ER Stress Sensor XBP1 Controls Anti-tumor Immunity by Disrupting Dendritic Cell Homeostasis. Cell. 2015; 161:1527–38. 10.1016/j.cell.2015.05.025. 26073941 PMC4580135

[30] Yamaguchi H , Wang HG . CHOP is involved in endoplasmic reticulum stress-induced apoptosis by enhancing DR5 expression in human carcinoma cells. J Biol Chem. 2004; 279:45495–502. 10.1074/jbc.M406933200. 15322075

[31] Bahar E , Kim JY , Yoon H . Chemotherapy Resistance Explained through Endoplasmic Reticulum Stress-Dependent Signaling. Cancers (Basel). 2019; 11:338. 10.3390/cancers11030338. 30857233 PMC6468910

[32] Reita D , Pabst L , Pencreach E , Guérin E , Dano L , Rimelen V , Voegeli AC , Vallat L , Mascaux C , Beau-Faller M . Direct Targeting *KRAS* Mutation in Non-Small Cell Lung Cancer: Focus on Resistance. Cancers (Basel). 2022; 14:1321. 10.3390/cancers14051321. 35267628 PMC8909472

[33] Liu J , Kang R , Tang D . The KRAS-G12C inhibitor: activity and resistance. Cancer Gene Ther. 2022; 29:875–78. 10.1038/s41417-021-00383-9. 34471232

[34] Begum P , Goldin RD , Possamai LA , Popat S . Severe Immune Checkpoint Inhibitor Hepatitis in *KRAS* G12C-Mutant NSCLC Potentially Triggered by Sotorasib: Case Report. JTO Clin Res Rep. 2021; 2:100213. 10.1016/j.jtocrr.2021.100213. 34590053 PMC8474489

[35] Ryan MB , Fece de la Cruz F , Phat S , Myers DT , Wong E , Shahzade HA , Hong CB , Corcoran RB . Vertical Pathway Inhibition Overcomes Adaptive Feedback Resistance to KRAS^G12C^ Inhibition. Clin Cancer Res. 2020; 26:1633–43. 10.1158/1078-0432.CCR-19-3523. 31776128 PMC7124991

[36] Zhao Y , Murciano-Goroff YR , Xue JY , Ang A , Lucas J , Mai TT , Da Cruz Paula AF , Saiki AY , Mohn D , Achanta P , Sisk AE , Arora KS , Roy RS , et al. Diverse alterations associated with resistance to KRAS(G12C) inhibition. Nature. 2021; 599:679–83. 10.1038/s41586-021-04065-2. 34759319 PMC8887821

[37] Awad MM , Liu S , Rybkin II , Arbour KC , Dilly J , Zhu VW , Johnson ML , Heist RS , Patil T , Riely GJ , Jacobson JO , Yang X , Persky NS , et al. Acquired Resistance to KRAS^G12C^ Inhibition in Cancer. N Engl J Med. 2021; 384:2382–93. 10.1056/NEJMoa2105281. 34161704 PMC8864540

[38] TargetScanHuman 7.2. 2023. https://www.targetscan.org/vert_72/.

