# Supplementary material for: Transformation of immunosuppressive mtKRAS tumors into immunostimulatory tumors by Nerofe and Doxorubicin
Source: Oncotarget. 2023 Jul 1;14:688–99. doi: 10.18632/oncotarget.28467 (PMC10317071; doi:10.18632/oncotarget.28467)
Supplement: Supplementary file 1 [file oncotarget-14-28467-s001.pdf]

Transformation of immunosuppressive mtKRAS tumors into immunostimulatory tumors by Nerofe and Doxorubicin

SUPPLEMENTARY MATERIALS

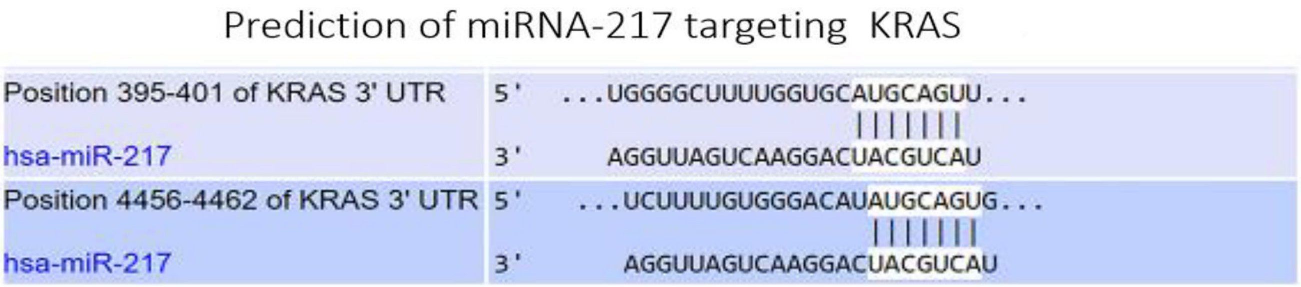

Supplementary Figure 1: Prediction for miR217 Predication was performed using TargetScan [38].

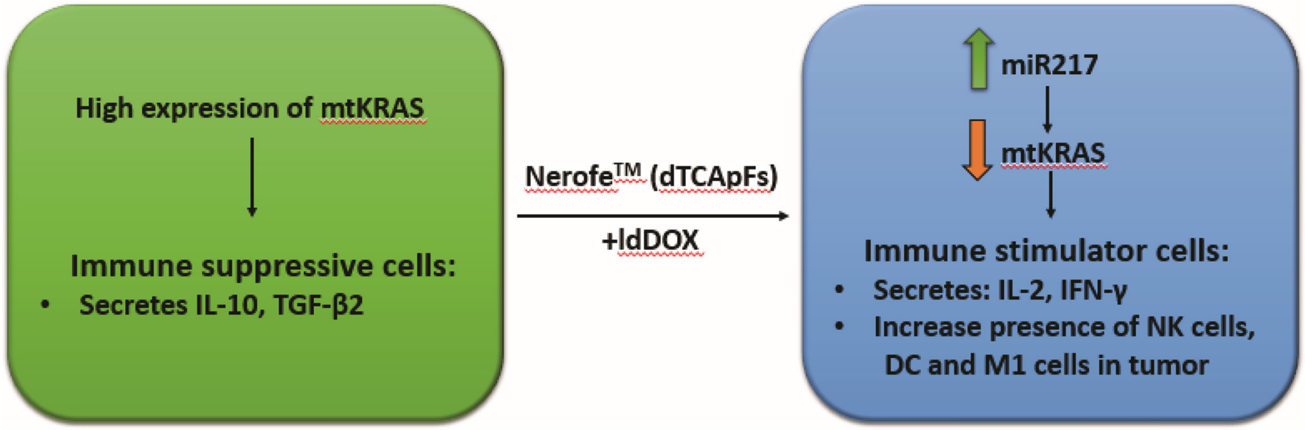

Supplementary Figure 2: Nerofe regulates immune characteristics of mtKRAS cells: High expression of mtKRAS stimulates immunosuppressive cells that secrete IL-10 and TGFb2. The combination of Nerofe and DOX downregulates mtKRAS via miR217, initiating secretion of IL-2 and IFN-γ, which results in increased tumor presence of NK cells, CD, and M1 cells.
